# Field evaluation of the safety, acceptability, and feasibility of early infant male circumcision using the AccuCirc device

**DOI:** 10.1371/journal.pone.0191501

**Published:** 2018-02-14

**Authors:** Robert C. Bailey, Irene Nyaboke, Mary Ellen Mackesy-Amiti, Erick Okello, Valentine Pengo, Betha Ochomo, Mary Emmaculate Auma, Simon Were, Stella Ojuok, Evelyne Adoyo, Mildred Adhiambo, Marisa R. Young, Rebeca M. Plank, Fredrick O. Otieno

**Affiliations:** 1 Division of Epidemiology and Biostatistics, School of Public Health, University of Illinois at Chicago, Chicago, IL, United States of America; 2 Nyanza Reproductive Health Society, Kisumu, Kenya; 3 Department of Gynecology and Obstetrics, Emory University, Atlanta, GA, United States of America; 4 Division of Infectious Diseases, Brigham and Women’s Hospital, Boston, MA United States of America; University of Ottawa Faculty of Medicine, CANADA

## Abstract

**Background:**

As countries scale up adult voluntary medical male circumcision (VMMC) for HIV prevention, they are looking ahead to long term sustainable strategies, including introduction of early infant male circumcision (EIMC). Although a number of devices for EIMC are prequalified by the World Health Organization, evaluation of additional devices can provide policy-makers and clinicians the information required to make informed decisions. We undertook a field evaluation of the safety and acceptability of the AccuCirc device in Kisumu County, Kenya.

**Methods:**

Procedures were performed by four trained clinicians in two public facilities. Participants were recruited from surrounding public health facilities through informational talks at antenatal clinics, maternity wards, and maternal neonatal child health clinics. Healthy infants ages 0–60 days, with no penile abnormality, without a family history of bleeding disorder, with current weight-for-age within –2 Z-scores of WHO growth standards, and whose mother was at least 16 years of age were eligible for EIMC. The procedure was performed after administration of a penile dorsal nerve block using 2% lidocaine and administration of Vitamin K. The mother was given post-operative instructions on wound care and asked to remain in the clinic with the baby for an observational period of one hour, during which a face-to-face questionnaire was administered.

**Results:**

Of 1259 babies screened, 704 were enrolled and circumcised. Median age of the infants was 16 days (IQR: 7–32.5) and of the mothers was 26 years (IQR: 22–30). Median time for the procedure was 19 minutes (IQR: 15–23). There were no serious adverse events (AE), and 20 (2.8%) moderate AEs, all of which were due to bleeding that required application of one to three sutures. There were 22 (3.8%) procedures in which the device did not fully incise the entire circumference of the foreskin and had to be completed using sterile scissors. 89.9% of mothers had knowledge of EIMC, but few (8.1%) had any knowledge of devices used for EIMC. Protection against HIV/AIDS was the most cited reason to circumcise a baby (65.3%), while the baby being ill (38.1%) and pain (34.4%) were the most cited barriers to uptake. 99% of mothers were “very satisfied” or “completely satisfied” with the procedure.

**Conclusions:**

This evaluation of the AccuCirc device is the largest to date and indicates that the device is safe and acceptable, achieving high levels of parental satisfaction. The AccuCirc device should be considered for WHO prequalification to increase options for safe and sustainable provision of EIMC.

## Introduction

Medical male circumcision (MMC) is a proven HIV prevention intervention, reducing the risk of heterosexual acquisition of HIV in men by 57–67% in three randomized controlled trials and in long-term follow-up studies [1–6]. The World Health Organization (WHO) and the Joint United Nations programme on HIV/AIDS (UNAIDS) endorsed scale-up of adolescent and adult voluntary medical male circumcision (VMMC) as part of comprehensive HIV prevention programs, and approximately 11.7 million circumcisions were achieved in 14 east and Southern African countries through 2015 [7]. As a few countries, including Kenya, have achieved or are approaching their original targets for total VMMCs, governments and donor agencies are considering whether and how best to transition from focusing on adolescent and adult circumcision to early infant male circumcision (EIMC), which might be more sustainable in the long term [8–10].

In 2010, the WHO published the “Manual for Early Infant Male Circumcision under Local Anaesthesia [11],” which included pre-qualification of the Mogen clamp, Gomco clamp and Plastibell devices for EIMC. Since that time, several studies and demonstration projects have employed the Mogen clamp [9,10,12–14], which is the only device currently approved by the Kenyan Ministry of Health for EIMC [15]. However, other devices are being field tested and are under consideration for adoption in several African countries.

**Fig 1:**
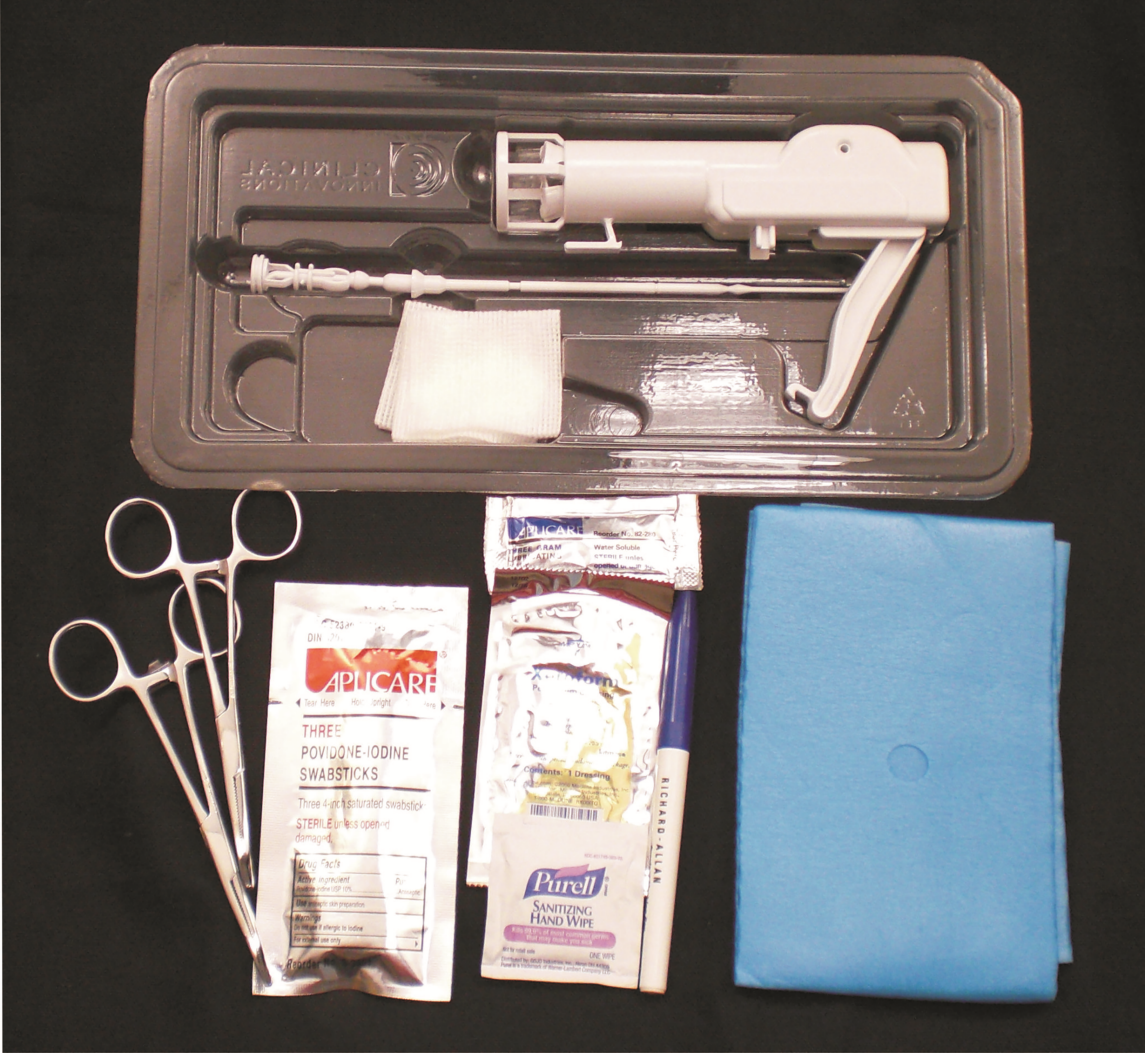
The self-contained disposable AccuCirc device kit.

A crucial consideration in assessing the effectiveness and acceptability of any EIMC device is safety. Although several studies have shown low rates of serious adverse events (AE) associated with EIMC [13,16–18], AE rates can be unacceptably high in some settings [19], and especially in the context of an elective procedure, AE rates should be minimized. There are rare but serious potential complications associated with all three devices currently on the WHO prequalified list of EIMC devices. Use of the Mogen clamp can result in partial or total amputation of the glans penis [11,16,20,21,22]. Migration of the Plastibell can result in necrosis of the glans and other injuries, and risk is increased if the incorrect size “bell” is used [11,13,23,24]. Mismatching the bell and base plate sizes of the Gomco clamp can result in laceration or amputation of the glans [11,20,24]. The potential complications of these three devices are inherent in their design. The recently developed AccuCirc device has several features designed to improve safety over other devices. It is a single-use disposable device designed to protect the glans from laceration or amputation. It consists of a shielding ring and a single-action clamp that contains a circular blade. The clamp is applied and activated to deliver a circumferential, hemostatic crush while simultaneously incising the foreskin. Its retractable blade cannot be reused and no part of the device is retained on the infant, unlike some tourniquet-type devices. In addition to possible safety advantages, the AccuCirc can simplify supply chain management by eliminating the need for instrument processing, since it comes in a sterile package containing everything necessary (except anaesthesia and gloves) to perform an EIMC [25]. The self-contained circumcision kit includes the foreskin probe/shielding ring, single-action clamp and blade, fenestrated drape with adhesive backing, surgical marking pen, hemostats, sanitizing wipe, iodine swab sticks, lubricating jelly, petrolatum dressing, and gauze (Fig 1). The kit is completely disposable.

Evaluations of specialized devices for EIMC are important to provide programs with the information required to make informed decisions about which devices could meet specific local needs. The WHO male circumcision device prequalification process is designed to assist program managers and sponsors supporting expansion of circumcision programs in making decisions about which devices are safest and most appropriate for integration with comprehensive HIV prevention programs [26]. The AccuCirc has previously been the subject of evaluation in Botswana [27] and Zimbabwe [28,29,30]. We here report the results of a field evaluation of the AccuCirc device to contribute to the WHO prequalification process and to assess the acceptability, safety and feasibility of introducing the device for EIMC in a traditionally non-circumcising community in sub-Saharan Africa where successful adult VMMC programs have been widespread since 2008.

## Methods

The study was carried out at the University of Nairobi, Illinois, and Manitoba (UNIM) Research and Training Centre (URTC) in Kisumu and the Ahero Sub-County Hospital (ACH) in Ahero by the Nyanza Reproductive Health Society (NRHS) in collaboration with the University of Illinois at Chicago (UIC) and the Brigham and Women’s Hospital (BWH).

### Training

Prior to the onset of the study, training was provided to study clinicians (clinical officers and nurses) all of whom had previous experience with EIMC using the Mogen clamp. The training curriculum was developed based on the WHO “Manual for Early Infant Male Circumcision under Local Anaesthesia [11],” as well as materials and protocols developed by the manufacturer and Dr. Rebeca Plank, who had previously undertaken a field evaluation of the AccuCirc in Botswana [27]. The training curriculum required eight hours of classroom-based learning followed by practicing the procedure on a model, witnessing at least two EIMC procedures, performing 10 procedures under supervision and being judged competent by a trainer. Two of the trainees were trained to be trainers after they had performed at least an additional 10 procedures using the AccuCirc device under the direction of the trainer.

### Recruitment and enrolment

Members of the study team identified health facilities in the Kisumu and Ahero catchment areas and gave informational presentations to all staff at each facility. The presentations included information about the risks and benefits of both adult and early infant male circumcision, different methods and devices used in EIMC, and the design and objectives of the AccuCirc study. Staff at each facility were requested to disseminate information about EIMC to pregnant mothers, as well as new mothers and fathers of a male. Study staff as well as some staff at the facilities actively identified and recruited eligible mothers at the antenatal clinics (ANC) and visited maternity wards and post-natal clinics to speak directly with mothers about the risks and benefits of EIMC and the possibility of participating in the study. Mothers who requested time to consult other relatives or their spouse were given time to do so and, when requested, the study staff also met with the relatives/spouse and explained EIMC. Those who wished to have their infants circumcised were referred to URTC or ACH depending on location. Once at the study clinic, they were provided with additional information on EIMC. Parents were given the option of participating in the study, in which the AccuCirc was used, or having EIMC performed as part of the national VMMC program using the Mogen clamp. Those opting to join the study provided written informed consent and their infants were screened for eligibility.

Subjects were recruited between February 2, 2015 and July 21, 2016. Eligibility criteria were: 1) male infant born within the study catchment area; 2) ages 24 hours to 60 days; 3) ability to attend scheduled study follow-ups; 4) provision of written informed consent by at least one parent or guardian; 5) no evidence of neonatal infection/sepsis or other current illness; 6) no penile abnormality that might require reconstructive surgery in the future; 7) no family history of bleeding disorder; 8) estimated infant gestational age ≥37 weeks; and 9) current weight-for-age for boys of at least -2 Z-scores according to WHO growth standards [31]. This last criterion meant that minimum weight for any baby was 2500 grams.

### The procedure

Following eligibility determination, the infants underwent a complete head-to-toe physical examination to rule out conditions that could preclude them from EIMC. To minimize risk of bleeding, all infants received vitamin K (1 mg) approximately one hour before the procedure. The area was prepped and draped in accordance with sterile procedures. Local anaesthesia was achieved by the use of 2% lidocaine dorsal penile nerve block with a maximum dose of 0.15 mg/kg. Anaesthesia was assessed by pinching the foreskin using an artery forceps 15 to 30 minutes following administration. Once no signs of pain were detected, AccuCirc circumcision was performed as previously described [27, 30]. Following circumcision, the wound was dressed with the parent observing. The parent was given instructions on how to care for the wound at home and instructed to return in three days. The parent was requested to remain in the clinic for one hour after completion of the procedure, at which time the infant was reviewed before discharge to home.

### Interview

During the hour that the parents and baby remained in the clinic for observation, a research assistant administered a questionnaire in either English, Kiswahili or DhoLuo to the mother to assess demographics, birth history, prenatal care and delivery, knowledge about EIMC, source(s) and timing of information about EIMC, beliefs and attitudes about MMC and EIMC, reasons for accepting EIMC, father’s role in decision-making, knowledge of EIMC devices and the role of devices in decision-making about EIMC (S1 Questionnaire). Only mothers participated in the interview. Each face-to-face interview took approximately 35 minutes.

Following the interview, the baby was re-assessed and the mother was again provided with instructions on wound dressing, detection and management of bleeding, infection or any abnormalities, and was given emergency contact information, which included a 24-hour hotline. The mother was encouraged to call the emergency numbers or to come to the clinic if she had any concerns or detected any unanticipated events between scheduled visits.

### Follow-up

The first 50 participants were followed up at 24 hours, three days, one week, and four weeks following the procedure. The subsequent 654 participants were scheduled for follow up at three days post-procedure only. All mothers were asked to bring their infants to the clinic at any time between scheduled visits or any time after the last scheduled visit if they had any concerns. During follow-up visits the clinician conducted a physical examination of the infant, including inspection of the circumcision site, and asked the parent if she had any concerns about the procedure or progress of the wound. In this paper we do not report the first 50 participants separately. We report the results of only the Day 3 post-procedure visit or of any unscheduled visits for the full sample of 704 infants and 700 mothers.

### Measures

Safety was measured by the number of moderate and severe adverse events (AEs). The primary EIMC-related AEs assessed were categorized as bleeding, infection, hematoma, inadequate or excessive skin removal, or penile injury (to the glans, urethra, or shaft). We assessed EIMC acceptability by the proportion of mothers who reported being satisfied with the procedure, and who expressed willingness to adopt EIMC for a future son. Other measures of interest included: median surgical time and parental satisfaction with AccuCirc EIMC services.

### Statistical analyses

All data were entered by the clinician or the research assistant on paper forms and entered into a REDCap database by the data manager. All statistical analyses were conducted using Stata SE version 13 (StataCorp, College Station, Texas, USA). We summarized characteristics of parents and infants, including socio-demographic characteristics, father’s reported circumcision status, and knowledge of circumcision and HIV. To evaluate the safety of the procedure, we calculated the proportion of procedures associated with AEs with 95% confidence intervals (CI). We estimated that the 95% CI around an AE rate of 0.02 would be 0.011 to 0.033 given a sample size of 700 circumcisions. 2% was chosen based on the historical proportion of males who experience an AE with surgical MC [1–3] and because it has been applied previously as the benchmark for a non-inferiority trial comparing the AccuCirc device and the Mogen clamp (29).

### Ethical considerations

The study was approved by the Maseno University Ethics Review Committee, the Brigham and Women’s Hospital/Partners Human Research Committee Institutional Review Board and the University of Illinois at Chicago Institutional Review Board. We obtained written informed consent from the infant’s mother and verbal consent from the infant’s father (if available) before enrolment. Following the circumcision visit, mothers received KES 300 (approximately $3US) for transportation and a pack of disposable infant diapers (6 pcs) for every follow-up visit.

## Results

**Fig 2:**
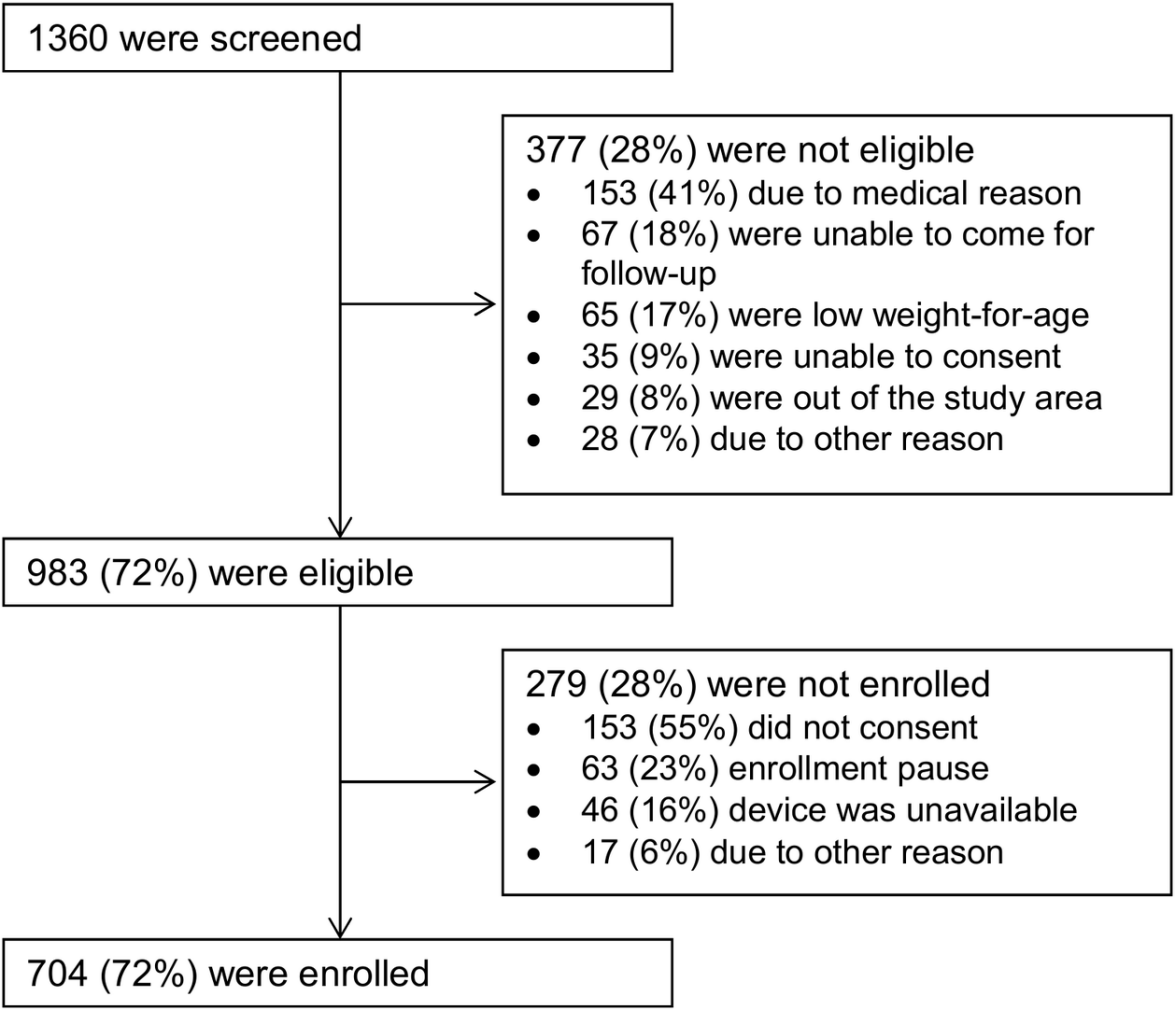
Screening and enrolment of infants in the AccuCirc evaluation study.

We screened a total of 1,360 infants of whom 377 (28%) were ineligible (Fig 2). The most prevalent reasons for ineligibility were health related reasons (41%), unable to come for follow-up (18%), low weight-for-age (17%), parent unable to consent (9.0%) and living out of the area (8%). There were 983 infants eligible for enrolment. Of these, 279 (28%) were not enrolled either because the correct size of the device was not available at the time of screening (n = 46, 16%), enrolment was paused at the time of screening (n = 63, 23%), or the parent declined to consent to be in the study (n = 153, 55%). Most of this last group did not want to be obligated to return for follow-up, as required in the study protocol, and they opted for their baby to be circumcised using the Mogen clamp as part of the national VMMC program. Therefore, 704 infants born to 700 mothers–there were 4 sets of twins—were enrolled in the study. Of note is that 218 babies, or 16% of all those screened, were ineligible for health reasons or due to low weight-for-age. This may indicate the proportion of babies who would not be eligible under a non-research setting.

The median age of the infants was 16 days with an interquartile range (IQR) of 7 to 32.5 days, while the mothers’ median age was 26 years (IQR: 22–30). Parents were predominantly Luo with 79.7% and 85.6% of mothers and fathers being Luo, respectively. The large majority (80.3%) of the mothers were married or living as married; 42.1% had some primary or no education, and 24.1% had a post-secondary education. Twenty percent were employed. Almost half (49.4%) reported their religion as Protestant, 21.9% Catholic, 12.9% Seventh Day Adventist, 7.4% Muslim, and 8.4% another or no religion. Mothers reported that 68.3% of the fathers of the babies were circumcised with 54.4% of these circumcised by a medical practitioner and 11.7% by a traditional practitioner; 33.9% of mothers were unsure of the method of circumcision (Table 1).

**Table 1:** AccuCirc for early infant male circumcision in Nyanza, Kenya: characteristics of mothers and fathers based on mother interviews (n = 700).

	n	%
Age (years)		
18–19	68	9.7
20–24	230	32.9
25–29	214	30.6
30+	188	26.9
Marital status		
Living with spouse/partner	562	80.3
Not living with spouse/partner	59	8.4
Single, widowed, separated or divorced	78	11.1
Refused to answer	1	0.1
Education		
Primary or less (0–8 years)	295	42.1
Any secondary (Form 1–4)	236	33.7
Post-secondary	169	24.1
Employment		
No	559	79.9
Yes	141	20.1
Income past month (Kenyan shilling)		
None	148	21.1
<2000	130	18.6
2000–4999	92	13.1
5000–9999	73	10.4
10000–25000	67	9.6
>25000	35	5.0
Don’t know/ Refused to answer	155	22.1
Religion		
Protestant	346	49.4
Catholic	153	21.9
Seventh Day Adventist	90	12.9
Muslim	52	7.4
Other	59	8.4
Parity		
One	189	27.0
Two	231	33.0
Three or more	280	40.0
Place of delivery		
Home	21	3.0
Hospital/clinic	672	96.0
Other	7	1.0
Previous son circumcised		
No	155	22.1
Yes	144	20.6
No previous son	401	57.3
Father's ethnicity		
Luo	599	85.6
Other	100	14.3
Refused to answer	1	0.1
Father is circumcised		
No	202	28.9
Yes	478	68.3
Don't know	20	2.9
Father circumcised by		
Medical practitioner	260	54.4
Traditional practitioner	56	11.7
Don't know	162	33.9
Mother’s HIV results last tested		
HIV-negative, prenatal test	411	58.7
HIV-negative, earlier test	133	19.0
HIV-positive/Infected	141	20.1
Don’t know/Not sure	1	0.1
Refused to answer	14	2.0
Perceived HIV status of the baby		
HIV-negative/Uninfected	539	77.0
HIV-positive/Infected	15	2.1
No opinion / Don’t know	146	20.9
Perceived HIV status of the father		
Sure he is HIV-negative	459	65.6
Sure he is HIV-positive	77	11.0
Think he is HIV-negative	47	6.7
Think he is HIV-positive	3	0.4
Don’t know/ Refused to answer	114	16.3

All but 14 (2.0%) of the mothers agreed to disclose their HIV status; just one mother said that she did not know her serostatus. Twenty percent reported that they were HIV-positive and 11.0% said that they were sure that the father of the baby was HIV-positive, with 16.3% of mothers either declining to answer the question or reporting that they did not know the father’s serostatus. Two percent of mothers perceived their infant to be seropositive, 77.0% perceived him to be negative, and 20.9% said that they did not know or were not sure (Table 1).

### The procedure

The AccuCirc device comes in two diameters: 1.1 cm and 1.3 cm. There were approximately equal numbers of each device size used: 342 (48.6%) with 1.1 cm and 362 (51.4%) with 1.3 cm. The smaller device size was used more often than the larger size for babies younger than 14 days and for babies lighter than 4000 g. There was no clear cut-off in terms of the baby’s age or weight when employing one size or the other. For example, the smaller size was used in as many as 18 (2.6%) babies in the 42–60 day age range and 16 (2.3%) in the 5000–6500 g weight range, and the larger size was used in 95 (13.5%) babies under 14 days and 106 (15.1%) of babies under 4000 g. In other words, both device sizes had to be available to enable accommodation of all baby ages and weights.

Among 704 procedures performed, the median time from when the baby was placed on the restraining board to when the procedure was completed and the baby handed to the mother was 19 minutes (IQR: 15–23). The median time from when the device was placed to when the foreskin and device were removed was six minutes (IQR: 5–6). This included five minutes of waiting time after the device was placed to allow for hemostasis. In some cases, when the baby was older and heavier, upon the judgement of the clinician, the device was left on the penis for an additional one to five minutes to allow more time for hemostasis.

### Adverse events and incomplete cuts

Among the 704 procedures, there were no serious AEs. There were 20 moderate AEs, or a rate of 2.8% (95% CI: 1.7%-4.4%), all of them due to bleeding and all managed with the application of one to three sutures. The baby was observed for 90–120 minutes to ensure that no additional bleeding occurred before he was allowed to go home. None of the cases required hospitalization, and all the wounds healed with no permanent sequelae. Because the clinicians felt that bleeding was more common among babies older than four weeks, we examined the association between age of the baby and the odds of bleeding. The AE rate among those babies older than 30 days (4.1%) was greater than among those 30 days or younger (2.4%), but the difference was not statistically significant (OR = 1.76; 95% CI: 0.61–4.76; *p* = 0.22). Notably, there were no infections.

In addition to the AEs reported above, there were also a number of device related events that the practitioners experienced when using the AccuCirc device. In 22 cases (3.1%) the AccuCirc device did not make a complete cut around the full 360 degrees of the foreskin. Most of these cases (16/22) were easily managed by cutting the small amount of remaining tissue with sterile scissors or a scalpel. However, in six cases the cutting resulted in bleeding that could not be staunched with pressure, and application of one to three sutures was necessary. These six cases have been included among the 20 moderate AEs reported above. Because the practitioners felt that an incomplete cut was more likely when the baby was larger, perhaps because his foreskin is thicker and less likely to be fully severed by the device blade, we examined the association of incomplete cuts with age. There was a trend toward a higher proportion of babies who were older than 30 days of age experiencing an incomplete cut (5.1%) compared to those 30 days or younger (2.4%), (OR = 2.22; 95% CI: 0.84–5.71; *p* = 0.06).

The study team discussed the issue of incomplete cuts with the manufacturer during the course of the study. After some discussion, the manner in which the arm of the device was depressed was modified to make it one strong continuous motion. This modification in technique reduced the rate of incomplete cuts from 3.1% to 1.9%, which is not significant due to low power to detect a difference.

### Male circumcision knowledge and decision making

Almost all mothers (96.4%) reported prior knowledge of adult MMC. Previous knowledge of EIMC was also high (89.9%) with the main sources of information being a health provider at a health facility (39.9%) and a health care worker in the community (20.6%), with friends/neighbors (12.7%) and family members (6.7%) also being sources of information. Public media (radio, newspaper, television) and posters and brochures were cited as sources of information by only 6.7% of mothers (Table 2). Nearly two-fifths (38.7%) of mothers learned about EIMC in maternity on the day of or the day after delivery. More than a quarter (27.3%) received information about EIMC before they were pregnant, 16.1% while they were pregnant, and 7.7% at a time later than around the birth of the baby. Regarding knowledge about EIMC devices, 82.4% had never heard of EIMC devices before they came to the clinic, and only 8.1% had ever heard the names of any devices, with the Mogen clamp and the AccuCirc as the most cited (Table 2).

**Table 2:** AccuCirc for early infant male circumcision in Nyanza, Kenya: medical male circumcision information and opinions from mother interviews (n = 700).

	n	%
Previously received information about MMC		
No	25	3.6
Yes	675	96.4
Previously received information about EIMC		
No	71	10.1
Yes	629	89.9
Sources of information on EIMC		
Health provider at a health facility	279	39.9
Health care worker in community	144	20.6
Friend(s)/Neighbor(s)	89	12.7
Family member(s)	47	6.7
Media (radio, newspaper, television)	30	4.3
Poster or brochure	17	2.4
School or university	8	1.1
Workshop or baraza	4	0.6
Others	39	5.6
When information was received		
Before this pregnancy	191	27.3
During this pregnancy	113	16.1
At delivery/Day after delivery	271	38.7
At a later time /Other	54	7.7
Not applicable	71	10.1
Heard of different devices?		
No	577	82.4
Yes	57	8.1
Yes, but does not know names	66	9.4
Heard of:		
Mogen clamp	34	4.9
Accucirc	39	5.6
Gomco	1	0.1
Plastibell	1	0.1
PrePex	6	0.9
Shang Ring	2	0.3
Son will be at risk of HIV infection		
No risk	4	0.6
Little risk	575	82.1
Some risk	70	10.0
High risk	7	1.0
Don't know /Not sure	44	6.3
Best age for male circumcision		
Birth to 60 days	664	94.9
Older than 60 days but <1 year old	3	0.4
1 to 9 years old	4	0.6
10 to 17 years old	12	1.7
18 years or older	4	0.6
Any age	3	0.4
Don’t know/Not sure	10	1.4
Reasons to circumcise a baby*		
Protection against HIV/STIs	659	94.1
Less pain than later	643	91.9
Penile hygiene	642	91.7
Protection against UTI	640	91.4
Safer than later	587	83.9
Improved cosmetic appearance	350	50.0
Religious reason	44	6.3
Cultural reasons	23	3.3
Most important reason to circumcise a baby		
Protection against HIV/STIs	457	65.3
Protection against UTI	28	4.0
Penile hygiene	22	3.1
Improved cosmetic appearance	0	0.0
Less pain than later	93	13.3
Safer than later	18	2.6
Religious reason	26	3.7
Cultural reasons	5	0.7
Other	49	7.0
Reasons not to circumcise a baby*		
The baby is unwell	382	54.6
Pain	365	52.1
Bleeding	310	44.3
Infection	273	39.0
Injury to the penis	256	36.6
The father is against it	175	25.0
The mother is unwell	89	12.7
Death from circumcision	45	6.4
It is against cultural tradition	20	2.9
It is better to wait	17	2.4
No reason not to circumcise	73	10.4
Most important reason not to circumcise a baby		
The baby is unwell	267	38.1
Pain	241	34.4
Bleeding	37	5.3
The father is against it	30	4.3
Infection	21	3.0
Injury to the penis	17	2.4
Death from circumcision	12	1.7
The mother is unwell	2	0.3
No reason not to circumcise	73	10.4
Who should participate in decision*		
Both mother and father equally	650	92.9
Mother of infant	33	4.7
Father of infant	23	3.3
Health care professionals	500	71.4
Other relatives	119	17.0
Friends	17	2.4
Traditional leaders	3	0.4
Most important decision maker		
Both mother and father equally	580	82.9
Mother of infant	57	8.1
Father of infant	53	7.6
Health care professionals	9	1.3
Other relatives	1	0.1
Talked with father about circumcision		
No	110	15.7
Yes	588	84.0
Don't know /Not sure	2	0.3
Was father in favor of circumcision		
Against	17	2.9
For/In favor of	568	96.6
Don't know /Not sure	3	0.5
Can you decide on your own		
No	327	46.7
Yes	372	53.1
Refused to answer	1	0.1
Circumcision is viewed favorably by friends and family		
Strongly disagree	6	0.9
Disagree somewhat	9	1.3
Agree somewhat	107	15.3
Strongly agree	567	81.0
Refused to answer	1	0.1
Don't know /Not sure	10	1.4

* Participant could endorse multiple categories

When asked the best age at which to circumcise, almost all the mothers (94.9%) said within the first 60 days of life and 12 mothers (1.7%) said between ages 10 and 17 years. Regarding the reasons to circumcise a baby, protection against HIV/STIs, less pain, penile hygiene and protection against urinary tract infections (UTI) were all cited. When asked to choose the most important reason to circumcise a baby, protection against HIV/STIs was by far the most frequent response (65.3%), with less pain than at an older age being a distant second (13.3%). Conversely, the baby being ill (38.1%) and pain during circumcision (34.4%) were the primary reasons given for not circumcising an infant.

Regarding who should participate in decision making, 82.9% of the mothers felt that both parents should be equal parties to the EIMC decision, with 8.1% saying the mother was most important and 7.6% saying the father was most important. Notably, only 1.3% of mothers felt that the health care provider was the most important decision-maker. When asked if she could make the EIMC decision on her own, 53.1% of mothers said that they could, yet 588 (84.0%) of the mothers said that they had consulted the father of the baby before circumcision. According to the mothers, 568 fathers (96.6%) who were consulted were in favour of EIMC and just 17 (2.9%) were against. Not having contact with the father of the baby or not wanting to consult him were the most common reasons (76%) given by the 110 women who did not talk with the father about the EIMC decision.

To get a sense of the mothers’ perceptions of how circumcision was viewed in their community, we asked mothers their level of agreement with the statement, “circumcision is viewed favourably by your friends and family.” The great majority (81.0%) strongly agreed, 15.3% somewhat agreed, and just 2.2% either strongly or somewhat disagreed, with 1.4% saying they didn’t know or were not sure.

### Mothers’ satisfaction

Mothers’ levels of satisfaction with the procedure were very high (Table 3). 99% of mothers were either “very satisfied” or “completely satisfied” with the outcome of the procedure, and 98.3% said that it was “very likely” that they would have their next baby boy circumcised. Similarly, 98.4% said that they were “very likely” to recommend the procedure to others.

**Table 3:** Mothers’ satisfaction (n = 699 mothers; 703 infants).

	n	%
Mother's satisfaction with the circumcision procedure		
Unsatisfied	1	0.1
Somewhat satisfied	5	0.7
Very satisfied	17	2.4
Completely satisfied	676	96.7
Mother's satisfaction with the outcome of the circumcision^1^		
Unsatisfied	3	0.4
Somewhat satisfied	4	0.6
Very satisfied	27	3.8
Completely satisfied	669	95.2
Mother's satisfaction with the written care Instruction		
Unsatisfied	1	0.1
Somewhat satisfied	6	0.9
Very satisfied	10	1.4
Completely satisfied	682	97.6
Mother's likelihood of having their next baby circumcised before 60 days of age		
Very unlikely	6	0.9
Somewhat unlikely	2	0.3
Somewhat likely	4	0.6
Very likely	687	98.3
Mother's likelihood of recommending EIMC (done up to 60 days of life) to others		
Very unlikely	6	0.9
Somewhat unlikely	1	0.1
Somewhat likely	4	0.6
Very likely	688	98.4

Note: One evaluation was missing

1. Mothers wuth twins responded for each infant separately

## Discussion

The purpose of this field evaluation of the AccuCirc device was to assess the safety, feasibility and acceptability of the device in a traditionally non-circumcising community and, thereby, contribute to the WHO process for prequalification of circumcision devices [26]. In performing 704 circumcisions among baby boys ages 0–60 days, we observed no serious adverse events. There were 20 moderate AEs (2.8%), all of them cases of bleeding that required application of one to three sutures. Parental satisfaction with the procedure was very high (99%) and 98% said that they would recommend EIMC to others. The level of acceptability was high. Among those who were eligible for the procedure, 28% did not get circumcised, but non-enrolment was generally related to unwillingness to participate in an experimental study, not to lack of willingness for the baby to be circumcised. Due to our recruitment procedures, which included general health talks and community mobilization, we cannot determine the proportion of parents who might have been exposed to demand creation activities who eventually volunteered to participate in the study. In our previous study in the region, we estimated uptake of EIMC to be 26% of baby boys born in the area [32], a rate higher than reported in other East and southern African countries [32] and higher than the 11% achieved during an AccuCirc evaluation in Zimbabwe [30].

There have been two previous evaluations of the AccuCirc device for EIMC conducted in sub-Saharan Africa, both with smaller sample sizes than this study [27, 30]. The single arm evaluation conducted among 151 infants up to age 28 days in Botswana by Plank and colleagues observed no major AEs and only one moderate AE which involved bleeding and was resolved 30 minutes after administration of vitamin K1 [33]. As in our study, Plank et al. [27] observed procedures in which the AccuCirc device did not achieve a complete cut around the entire penile circumference, necessitating manual completion of the cut using sterile surgical scissors. A randomized non-inferiority trial conducted in Zimbabwe compared the safety of 50 Mogen clamp and 100 AccuCirc procedures [30]. Among the AccuCirc procedures, two moderate AEs occurred: one case of excess skin removal, which required application of hydrocortisone cream, and one case of inadequate skin removal, which warranted corrective surgery. This Zimbabwe study also experienced one case of incomplete incision, which occurred during training and was easily managed by completing the cut with sterile scissors. These two studies of the AccuCirc device combined with ours indicate that rates of moderate AEs using the AccuCirc may be higher than rates using the Mogen clamp. Importantly, however, due to incorporation of a shielding ring in the AccuCirc device, there is no risk of laceration injury to the glans penis; whereas with the Mogen clamp injuries to the glans and even amputation of the glans can occur [18,21,22,34]. Currently, the Kenyan government has approved use of only the Mogen clamp for its national EIMC program [15] and this seems to be the preferred device in use in most African EIMC programs. Because of its inherent design in which the glans is protected, the AccuCirc should be considered for use in national EIMC programs. In a recent study, providers in Kenya who had experience using both the Mogen clamp and the AccuCirc, unanimously expressed the view that the AccuCirc would be safest and most appropriate for a national EIMC program [35].

In addition to avoiding the possibility of an injury to the glans, the Accuirc has several other advantages over the Mogen clamp. The non-inferiority trial in Zimbabwe estimated that the unit costs of EIMC are greater using the Mogen compared to the AccuCirc device ($55.93US versus $49.53US), and their calculations did not include the costs of possible AEs, which might be greater when employing the Mogen due to their severity [28]. Most of the difference in costs between the devices is attributable to the AccuCirc coming in a pre-packaged kit that includes most of the consumable supplies required for an EIMC and, because AccuCirc is disposable, it does not require the sterilization facilities and supplies required for the Mogen clamp. In our study, we found the AccuCirc kit to be extremely convenient, requiring minimal purchasing and inventory of consumables, and no need for timely instrument disinfection processes. In a large EIMC program, simplification of supply chain management and the elimination of the need for reusable instrument inventory and sterilization will be significant advantages of the AccuCirc.

A drawback of the AccuCirc device for EIMC is the possibility that the device will fail to achieve a complete incision around the full circumference of the foreskin. In our study, 22/704 (3.1%) of procedures resulted in incomplete cuts, and over the three evaluations of the AccuCirc in sub-Saharan Africa conducted to date [27,3028,31], 25 of 919 (2.7%) have resulted in incomplete cuts. In the great majority of cases, these cause no harm, since they are easily managed by completing the cut using sterile scissors or a scalpel. However, in a few cases (n = 6), completing the cut can cause bleeding that may not be managed by applying pressure alone and may require one or more sutures. Midway through our study, we discovered that applying steady continuous pressure when activating the lever arm of the device reduced the risk of an incomplete cut, and this lesson should be incorporated into any training programs for use of the AccuCirc. In addition, the manufacturer should explore revising the device to minimize instances of incomplete cuts. An alternative would be to restrict EIMCs to infants younger than 30 days, since we found a trend toward a higher proportion of babies who were older than 30 days of age experiencing an incomplete cut (5.1%) compared to those 30 days or younger (2.4%). However, the 60-day window for achieving large numbers of EIMCs is already short; reducing it by half would adversely impact the efficacy of EIMC programs substantially. For example, 30% of our participants were older than 30 days.

The potential for incomplete cuts that may result in the need for sutures would restrict the use of the device to facilities with standard minor surgical instruments and personnel capable of applying sutures. However, this is the case for EIMC programs using any device [11].

### Limitations

Limitations of this study include our inability to assess the proportion of parents exposed to demand creation messages who ultimately sought EIMC services. This limitation is inherent to our recruitment activities that included general talks at ANC, at maternal neonatal child health clinics and at maternity wards, as well as training of personnel at numerous health facilities to educate women about EIMC and to assist with recruitment and referral to our study. These conditions are likely to be similar when EIMC programs are scaled up. Because some data were collected via face-to-face interviews, information bias, such as social desirability, is possible. This may be especially true regarding mothers’ levels of satisfaction, although the interviewers were not themselves clinicians and were not involved in performing procedures. Our study was not designed as a head-to-head comparison of EIMC using the Mogen clamp versus the AccuCirc device. One such trial has been published [30] and there are now sufficient results from studies using the Mogen clamp to assess differences in safety between the two devices. We have attempted to point out the advantages and disadvantages of using both devices based on our results and information available in the published literature.

## Conclusions

With a sample size of 704 infants ages 0–60 days, this is the largest study to date of EIMC using the AccuCirc device. The device appears to be safe and acceptable, achieving very high levels of satisfaction among parents of babies circumcised with the device. The AccuCirc should be considered for WHO pre-qualification to increase the options for safe and sustainable provision of EIMC.

## Supporting information

S1 Questionnaire Kenya AccuCirc study parental questionnaire English. (DOCX)
