# Supplementary material for: Field evaluation of the safety, acceptability, and feasibility of early infant male circumcision using the AccuCirc device
Source: PLoS One. 2018 Feb 14;13(2):e0191501. doi: 10.1371/journal.pone.0191501 (PMC5812570; doi:10.1371/journal.pone.0191501)
Supplement: S1 Questionnaire — (DOCX) [file pone.0191501.s001.docx]

|  | **Questionnaire – Parents** |
| --- | --- |
| **Date (dd/mm/yy): ____/____/____** | **Participant Number: _________________________**  **Site ID:_________** |

*[To be answered by the interviewer:]*

1. *Start Time (HH:MM 24-hour clock)*

| **:** |
| --- |

1. *Record geographic coordinates of location*

| ⁰ | ’ | ” | S |  |
| --- | --- | --- | --- | --- |

| ⁰ | ’ | ” | E |  |
| --- | --- | --- | --- | --- |

1. *Location of interview*

*🞎 1 = Home of baby*

*🞎 2 = Home of respondent (but not baby)*

*🞎 3 = NRHS / UNIM clinic*

*🞎 4 = Ahero sub-District Hospital*

*🞎 5 = Jaramogi Oginga Odinga Teaching and Referral Hospital*

*🞎 6 = Other (specify): ________________________________*

1. *Timing of interview:*

*🞎 1 = At the time of circumcision (≤ 60 days of age)*

*🞎 2 = OPV-2 visit (> 60 days of age)*

**NOTE: If mother has already completed this questionnaire she should not complete it again.**

**Part 1: Demographic Information**

*[Read*] I am going to start by asking you some questions about yourself, the baby and the father of the baby. As I explained in the consent form, you are free to refuse to answer any questions you don’t want to answer or are not comfortable answering and you can end the interview at any time. Are you ready to begin?

1. What is your own date of birth? *[dd/mm/yy]* ______/______/______
2. How old are you now? ___________ years old
3. What is your son’s date of birth? *[dd/mm/yy]* ______/______/______
4. What is your Ethnic origin? *[check one]*

🞎 1 = Luo

🞎 0 = Other (specify): ________________________________

1. What is the Ethnic origin of the father of the baby? *[check one]*

🞎 1 = Luo

🞎 0 = Other (specify): ________________________________

🞎 2 = Not sure

1. What county do you currently reside in (spend the most nights sleeping in)? *[check one]*

🞎 1 = Kisumu County

🞎 2 = Siaya County

🞎 3 = Homa Bay County

🞎 4 = Kakamega County

🞎 5 = Other (specify): ______________________________

1. What is your current marital status? *[check one]*

🞎 1 = Having a partner but not legally married, and not living together

🞎 2 = Living with a regular partner but not legally married

🞎 3 = Legally married, but not living with husband

🞎 4 = Legally married, living with husband

🞎 5 = Was legally married but now separated or divorced

🞎 6 = Widowed

🞎 7 = Single (no regular partner)

🞎 8 = Other (please specify): ___________________________________

1. Other than you, how many wives does the father of the baby currently have? *[write number in box]*

|  | number of wives other than you |
| --- | --- |

1. What is the circumcision status of the father of the baby [*interviewer to show mother the illustration used at the end of the consent form*]? *[check one]*

🞎 1 = Circumcised

🞎 0 = Uncircumcised

🞎 2 = Not sure

1. If **CIRCUMCISED**, when in his life was the father of the baby circumcised? *[check one]*

🞎 1 = As a baby

🞎 2 = As a child

🞎 3 = As a teenager

🞎 4 = As an adult

🞎 5 = Not sure

1. If **CIRCUMCISED**, who circumcised him? *[check one]*

🞎 1 = A clinician (nurse, doctor, clinical officer)

🞎 2 = A traditional circumciser

🞎 3 = Not sure

🞎 4 = Other (specify): ______________________________

1. If **CIRCUMCISED**, was the father of the baby circumcised by a clinician as part of the national Voluntary Medical Male Circumcision program?

🞎 = 1 Yes

🞎 = 0 No

🞎 = 2 Not sure

1. What is the highest level of school you completed? *[check one]*

🞎 0 = No level completed

-----------------------------------

🞎 1 = Class 1

🞎 2 = Class 2

🞎 3 = Class 3

🞎 4 = Class 4

🞎 5 = Class 5

🞎 6 = Class 6

🞎 7 = Class 7

🞎 8 = Class 8

-----------------------------------

🞎 9 = Form 1

🞎 10 = Form 2

🞎 11 = Form 3

🞎 12 = Form 4

-----------------------------------

🞎 13 = Post-graduate Certificate

🞎 14 = Post-graduate Diploma

🞎 15 = Post-graduate Degree

1. Are you currently employed? *[check one]*

🞎 1 = Yes

🞎 0 = No

1. In the past month, how many shillings have you earned from all sources? *[check one]*

🞎 1 = None

🞎 2 = < 2000

🞎 3 = 2000-4999

🞎 4 = 5000-9999

🞎 5 = 10000-25000

🞎 6 = > 25000

1. What is the main lighting source for your home? *[check one]*

🞎 1 = Candles / Kerosene lamps

🞎 2 = Solar power

🞎 3 = Mains electricity

🞎 4 = Other (specify):_________________________

1. What do you primarily use to cook food in your home? *[check one]*

🞎 1 = Firewood

🞎 2 = Charcoal stove (jiko)

🞎 3 = Kerosene stove

🞎 4 = Gas/electric cooker

🞎 5 = Other (specify):_________________________

1. What is your religion? *[check one]*

🞎 1 = Seventh Day Adventist

🞎 2 = Nomiya

🞎 3 = Protestant (specify which): ___________________________________

🞎 4 = Africa Independent Churches (eg: Roho, Legio Maria)

🞎 5 = Catholic

🞎 6 = Muslim

🞎 7 = Other (please specify):_________________________

🞎 8 = None

🞎 9 = Not sure

**FOR THOSE NOT INTERVIEWED AT HOME** *[for interviews conducted at home, please skip to Part II, question 23]*

1. How did you get here today? *[check all that apply]*

🞎 1 = On foot

🞎 2 = Bicycle taxi or motorcycle taxi (boda boda or piki piki)

🞎 3 = Tuk tuk

🞎 4 = Matatu

🞎 5 = Private car (taxi)

🞎 6 = Other (specify): ________________________________

1. How much did it cost to arrive here today? *[write number in box]*

|  |  |  | Kenya Shillings |
| --- | --- | --- | --- |

🞎 999 = Not sure

1. About how long did it take to travel here from your home today? *[Write number in box in minutes. Eg: 2 hours = 120 minutes.]*

|  |  |  | Minutes |
| --- | --- | --- | --- |

🞎 999 = Not sure

**Part 2: Birth history, prenatal care and delivery**

*[Read] Now I am going to ask you some questions about your son, other children you may have, and your pregnancy*

1. How many times have you given birth to a live baby including babies who are still alive and those who have died since being born alive, including this one [name]? *[write number in box]*

|  |  | Live Births |
| --- | --- | --- |

1. Of these *[number]* children, how many were sons, including this one [name]? *[write number in box]*

|  |  | Sons |
| --- | --- | --- |

1. Of these *[number]*sons, how many are/were circumcised, do not count this one [name]? *[write number in box]*

|  |  | Circumcised |
| --- | --- | --- |

1. Did you receive any antenatal care, that is, did anyone check on your health or the health of your baby when you were pregnant with *[name]*? *[check one]*

🞎 1 = Yes *[skip a; complete b through e]*

🞎 0 = No *[complete a; skip b through e]*

🞎 2 = Not sure *[skip to 27]*

1. If **NO**, why did you not receive antenatal care? *[check all that apply]*

🞎 1 = Too expensive

🞎 2 = Clinic too far away

🞎 3 = Wait is too long at the clinic

🞎 4 = No transportation

🞎 5 = Didn’t think I needed antenatal care

🞎 6 = Family/friends did not want me to go

🞎 7 = Health care worker did not want me to go

🞎 8 = Other (specify): __________________________________________________

1. If **YES**, where did you receive antenatal care? *[probe to identify each location, record all mentioned]*

🞎 1 = Your home

🞎 2 = Other home

🞎 3 = Ahero sub-District Hospital

🞎 4 = Jaramogi Oginga Odinga Teaching and Referral Centre

🞎 5 = Other Government hospital/clinic (specify): ____________________________

🞎 6 = Private hospital/clinic (specify): _____________________________________

🞎 7 = Other place (specify): _____________________________________________

1. If **YES**, who provided the care? *[probe to identify each person, record all mentioned]*

🞎 1 = Doctor/Clinical Officer

🞎 2 = Nurse/Midwife

🞎 3 = Traditional Birth Attendant

🞎 4 = Community Health Worker

🞎 5 = Other (specify): ____________________________________

1. If **YES**, about how many months pregnant were you when you first received antenatal care for this pregnancy? *[Write number in box]*

|  |  | Months |
| --- | --- | --- |

🞎 99 = Not sure

1. If **YES**, about how many visits did you have for antenatal care for this pregnancy? *[Write number in box]*

|  |  | Times |
| --- | --- | --- |

🞎 99 = Not sure

1. Do you know your HIV status? *[check one]*

🞎 1 = Yes *[go to a]*

🞎 0 = No *[go to 28]*

🞎 2 = Not sure *[go to 28]*

🞎 3 = Refused to answer *[go to 28]*

1. If **YES**, when was your most recent test for HIV? *[check one]*

🞎 1 = I was tested as part of pre-natal care during this most recent pregnancy

🞎 2 = I was tested as part of pre-natal care but during a previous pregnancy

🞎 0 = I was not pregnant the last time I got tested

🞎 3 = Not sure

🞎 4 = Refused to answer

1. If **YES**, are you willing to give your most recent results? *[check one]*

🞎 1 = Yes

🞎 0 = No

1. If **YES**, what were they? *[check one]*

🞎 1 = Positive

🞎 0 = Negative

🞎 2 = Not sure

1. Whether you have been tested or not, in your opinion, what is your HIV status? *[check one]*

🞎 1 = HIV positive

🞎 0 = HIV negative

🞎 2 = Not sure

🞎 3 = Refused to answer

1. Have you ever talked with the father of the baby about his HIV status? *[check one]*

🞎 1 = Yes

🞎 0 = No

🞎 2 = Not sure

🞎 3 = Refused to answer

1. Have you tested for HIV together with the father of the baby?

🞎 1 = Yes

🞎 0 = No

🞎 2 = Not sure

🞎 3 = Refused to answer

1. In your opinion, do you believe that your baby is HIV positive or HIV negative?

🞎 1 = Positive

🞎 0 = Negative

🞎 2 = No opinion

1. What do you think the HIV status of the father of the baby is? *[check one]*

🞎 1 = I think he is HIV positive

🞎 2 = I am sure he is HIV positive

🞎 3 = I think he is HIV negative

🞎 0 = I am sure he is HIV negative

🞎 4 = I am not sure if he is HIV infected or not

🞎 5 = Refused to answer

1. Where did you give birth to your last-born (THIS) son *[name]*? *[Check one. Probe to identify the location]*

🞎 1 = Your home *[if home, go to 29a]*

🞎 2 = Other home (specify) *[if home, go to 29a]*:___________________________________

🞎 3 = Ahero sub-District Hospital *[go to 30]*

🞎 4 = Jaramogi Oginga Odinga Teaching and Referral Hospital *[go to 30]*

🞎 5 = Other Government hospital/clinic (specify) *[go to 30]:*__________________________

🞎 6 = Private hospital/clinic (specify) *[go to 30]*: ___________________________________

🞎 7 = Other place (specify) *[go to 30]:*___________________________________________

1. If **YOUR HOME** or **OTHER HOME**, what are the reasons that you did not deliver at a hospital or health care facility? *[check all that apply]*

🞎 1 = Charges at the health facility

🞎 2 = Cost of transport to the facility

🞎 3 = Delivery came before I could reach the facility

🞎 4 = Comfort with the home environment

🞎 5 = Presence of family members/neighbors to support me

🞎 6 = Fear of health care workers

🞎 7 = Fear of possible mistreatment at facilities

🞎 8 = Fear of cesarean section

🞎 9 = Fear of death

🞎 10 = Told by TBA/health care provider not to go to hospital/health care facility

🞎 11 = Other (specify): ____________________________________

1. Who participated in the decision as to where you delivered *[name]*? *[check all that apply]*

🞎 1 = Myself mostly

🞎 2 = The father of the baby mostly

🞎 3 = Myself and the father equally

🞎 4 = Family member(s) *[specify relationship to respondent]* _________________________

🞎 5 = Traditional Birth Attendant

🞎 6 = Health care provider such as nurse, midwife, clinical officer, medical doctor

🞎 7 = Friends/neighbors

🞎 8 = Other (specify): ____________________________________

🞎 9 = Not sure

1. Who assisted with the delivery of your son *[name]*? Anyone else? *[Probe for the type(s) of person(s) and record all mentioned. If respondent says no one assisted, probe to determine whether any adults were present at the delivery]*

🞎 1 = Doctor/Clinical Officer

🞎 2 = Nurse/Midwife

🞎 3 = Traditional Birth Attendant

🞎 4 = Relative/Friend

🞎 5 = Other (specify): ____________________________________

🞎 6 = No one assisted

**Part 3: Circumcision**

*[Read]* Now I am going to ask you some questions about male circumcision.

1. Before today, had you received information about **ADOLESCENT/ADULT** male circumcision? *[check one]*

🞎 1 = Yes

🞎 0 = No *(skip to 37)*

🞎 2 = Not Sure *(skip to 37)*

1. If **YES**, where? *[check all that apply]*

🞎 1 = From a health care worker in the community (for health care worker at a clinic / hospital choose #12 below)

🞎 2 = Family member(s) (specify relationship to the respondent):_______________

🞎 3 = Friend(s)

🞎 4 = Radio

🞎 5 = Newspaper

🞎 6 = Television

🞎 7 = Workshop or baraza

🞎 8 = Poster or brochure

🞎 9 = School or University

-----------------------------------

🞎 At a hospital/clinic: (specify):______________________________

🞎 10 = Poster or brochure

🞎 11 = Group health talk

🞎 12 = Individual consultation with a health provider

🞎 13 = From staff from this “AccuCirc Study”

🞎 14 = Other (specify): _________________________________

-----------------------------------

🞎 15 = Other (specify): ________________________________________

🞎 16 = Not sure

1. Before this interview right now, have you ever been given information / did you know about **INFANT** male circumcision, that is, circumcising before 60 days of age? *[check one]*

🞎 1 = Yes

🞎 0 = No *(skip to 38)*

🞎 2 = Not Sure *(skip to 38)*

1. If **YES**, where? *[check all that apply]*

🞎 1 = From a health care worker in the community (for health care worker at a clinic / hospital choose #12 below)

🞎 2 = Family member(s) (specify relationship to the respondent [mother]):_____________________

🞎 3 = Friend(s)

🞎 4 = Radio

🞎 5 = Newspaper

🞎 6 = Television

🞎 7 = Workshop or baraza

🞎 8 = Poster or brochure (specify location):_________________________________

🞎 9 = School or University

-----------------------------------

🞎 At a hospital/clinic: (specify):__________________________________________

🞎 10 = Poster or brochure

🞎 11 = Group health talk

🞎 12 = Individual consultation with a health provider

🞎 13 = From staff from this “AccuCirc Study”

🞎 14 = Other (specify): __________________________________________

-----------------------------------

🞎 15 = Other (specify): ________________________________________________

🞎 16 = Not sure

1. If **YES**, when? *[check all that apply]*

🞎 1 = Before this pregnancy

🞎 2 = During this pregnancy but before delivery

🞎 3 = At delivery / later the day of delivery or day after delivery

Between delivery and now

🞎 4 = At the child welfare clinic a week or less after birth

🞎 5 = At a vaccination visit

🞎 6 = At a visit because baby was sick

🞎 7 = Other visit (specify):_______________________________________

🞎 8 = Today at this health facility, before hearing about the “AccuCirc Study”

🞎 9 = Only now from staff from the “AccuCirc Study”

🞎 10 = Other (specify): _________________________________________________

🞎 11 = Not sure

1. Before this interview, have you ever heard that there are different devices/methods used for circumcision? *[check one]*

🞎 1 = Yes

🞎 0 = No *(skip to 39)*

🞎 2 = Not Sure

1. If **YES or Not Sure**, which devices have you heard about? *[A: unprompted; B: read list of answers and check all that apply]*

**A B**

🞎 🞎 1 = Mogen Clamp

🞎 🞎 2 = AccuCirc

🞎 🞎 3 = Gomco

🞎 🞎 4 = Plastibell

🞎 🞎 5 = PrePex

🞎 🞎 6 = Shang Ring

🞎 🞎 7 = Any other device (specify) = __________________________

🞎 🞎 0 = Has not heard of specific devices

1. To what extent do you believe that your son *[name]* will be at risk of becoming HIV infected when he becomes a man? *[check one]*

🞎 3 = High risk of becoming infected

🞎 2 = Some risk of becoming infected

🞎 1 = Little risk of becoming infected

🞎 0 = No risk of becoming infected

1. In general, is male circumcision an acceptable practice to you? *[check one]*

🞎 1 = Yes

🞎 0 = No

🞎 2 = Not sure

1. In your opinion, what is the best age for male circumcision? *[check all that apply]*

🞎 1 = Birth to two months

🞎 2 = Older than two months but <1 year old

🞎 3 = 1 to 9 years old

🞎 4 = 10 to 17 years old

🞎 5 = 18 years or older

🞎 0 = There is no good age for male circumcision

🞎 6 = Any age is good for male circumcision

1. In general, are you for or against a baby being circumcised before he turns two months old? *[check one]*

🞎 1 = For

🞎 0 = Against

🞎 2 = Not sure

1. In your opinion, what are all the reasons to circumcise a baby boy?

*[A: DO NOT read list of answers –****check all that apply****. Probe 🡺 Any other reason?]*

*[B: Read list of answers –****check all that apply****]*

**A B**

- 🞎 1 = Protection against HIV/STI
- 🞎 2 = Protection against Urinary Tract Infection (UTI)
- 🞎 3 = Penile hygiene / cleanliness
- 🞎 4 = Improved cosmetic appearance of the penis
- 🞎 5 = Less pain than when done later
- 🞎 6 = It is safer than when done later
- 🞎 7 = Religious reason
- 🞎 8 = Cultural reasons
- 🞎 9 = There is no reason to circumcise a baby boy
- 🞎 10 = Not sure
- 🞎 11 = Other (specify): ________________________________________

1. Of those reasons, in your opinion, what is the most important reason to circumcise a baby boy? *[Read respondent’s answer[s] from previous question–****check only one****]*

🞎 1 = Protection against HIV/STI

🞎 2 = Protection against Urinary Tract Infection (UTI)

🞎 3 = Penile hygiene / cleanliness

🞎 4 = Improved cosmetic appearance of the penis

🞎 5 = Less pain than when done later

🞎 6 = It is safer than when done later

🞎 7 = Religious reason

🞎 8 = Cultural reasons

🞎 9 = There is no reason to circumcise a baby boy

🞎 10 = Not sure

🞎 11 = Other (specify): ________________________________________

1. In your opinion, what are all reasons not to circumcise a baby boy?

*[A: DO NOT read list of answers –****check all that apply****. Probe 🡺 Any other reason?]*

*[B: Read each answer –****check all that apply****]*

**A B**

🞎 🞎 1 = Pain

🞎 🞎 2 = Bleeding

🞎 🞎 3 = Infection

🞎 🞎 4 = Injury to the penis

🞎 🞎 5 = Death from circumcision

🞎 🞎 6 = Going against cultural tradition

🞎 🞎 7 = It is better to wait until the boy is older

🞎 🞎 8 = If the father is against it

🞎 🞎 9 = If the baby is unwell

🞎 🞎 10 = If the mother is unwell / tired after birth

🞎 🞎 11 = There is no reason not to circumcise a baby boy

🞎 🞎 12 = Not sure

🞎 🞎 13 = Other (specify): ________________________________________

1. Of those reasons, in your opinion, what is the most important reason not to circumcise a baby boy? *[Read respondent’s answer[s] from previous question–****check only one****]*

🞎 1 = Pain

🞎 2 = Bleeding

🞎 3 = Infection

🞎 4 = Injury to the penis

🞎 5 = Death from circumcision

🞎 6 = Going against cultural tradition

🞎 7 = It is better to wait until the boy is older

🞎 8 = If the father is against it

🞎 9 = If the baby is unwell

🞎 10 = If the mother is unwell / tired after birth

🞎 11 = There is no reason not to circumcise a baby boy

🞎 12 = Not sure

🞎 13 = Other (specify): ________________________________________

1. In your opinion, who are all the people who should participate in the decision about circumcision for a baby boy?

*[A: DO NOT read list of answers –****check all that apply****. Probe 🡺 Any other reason?]*

*[B: Read each answer –****check all that apply****]*

**A B**

🞎 🞎 1 = Mother of infant

🞎 🞎 2 = Father of infant

🞎 🞎 3 = Both of the infant’s parents equally

🞎 🞎 4 = Your own father

🞎 🞎 5 = Your own mother

🞎 🞎 6 = Your brothers or sisters

🞎 🞎 7 = Your uncle

🞎 🞎 8 = Your cousins

🞎 🞎 9 = Your father-in-law

🞎 🞎 10 = Your mother-in-law

🞎 🞎 11 = Your friends

🞎 🞎 12 = Traditional healer

🞎 🞎 13 = Traditional leaders

🞎 🞎 14 = Doctor / nurse / other health care professional

🞎 🞎 15 = Other *(please explain)*:

1. In your opinion, who is the most important person in the decision about circumcising your son?

🞎 1 = Mother of infant (respondent)

🞎 2 = Father of infant

🞎 3 = Both of the infant’s parents equally

🞎 4 = Your own father

🞎 5 = Your own mother

🞎 6 = Your brothers or sisters

🞎 7 = Your uncle

🞎 8 = Your cousins

🞎 9 = Your father-in-law

🞎 10 = Your mother-in-law

🞎 11 = Your friends

🞎 12 = Traditional healer

🞎 13 = Traditional leaders

🞎 14 = Doctor / nurse / other health care professional

🞎 15 = Other *(please explain)*:

1. Have you ever talked to the father of this baby *[name]* about circumcision for your son? *[check one]*

🞎 1 = Yes *[complete a and b and skip c]*

🞎 0 = No *[complete c only]*

🞎 2 = Not sure *[skip to 50]*

- 1. If **YES**, when? *[check one]*

🞎 1 = Before the baby was born

🞎 2 = Around the time of delivery or by the next day

🞎 3 = >1 day after delivery

- 1. If **YES**, was he for or against circumcision generally? *[check one]*

🞎 1 = For

🞎 0 = Against *[skip to 50]*

🞎 2 = Not sure *[skip to 50]*

- 1. If **YES,** was he for or against circumcising the baby **before two months**? *[check one then go to 50]*

🞎 1 = For

🞎 0 = Against

🞎 2 = Not sure

- 1. If **NO**, what are the reasons you did not talk about it? *[check all that apply]*

🞎 1 = I did not know circumcision was available

🞎 2 = No contact with the father

🞎 3 = I do not consult the father about the baby

🞎 4 = I did not want the boy to be circumcised

🞎 5 = I think the father will not want circumcision for the baby

🞎 5 = Not sure

🞎 6 = Other (specify): _________________________________________________

1. Have you consulted anyone else besides the father of the baby in deciding about whether to circumcise your son? *[check one]*

🞎 1 = Yes *[if yes, go to a]*

🞎 0 = No *[if no, skip to 51]*

1. If **YES**, who? *[****check all that apply****]*

🞎 1 = Mother of infant (respondent)

🞎 2 = Father of infant

🞎 3 = Both of the infant’s parents equally

🞎 4 = Your own father

🞎 5 = Your own mother

🞎 6 = Your brothers or sisters

🞎 7 = Your uncle

🞎 8 = Your cousins

🞎 9 = Your father-in-law

🞎 10 = Your mother-in-law

🞎 11 = Your friends

🞎 12 = Traditional healer

🞎 13 = Traditional leaders

🞎 14 = Doctor / nurse / other health care professional

🞎 15 = Other *(please explain)*:

1. Can you decide on your own whether or not to circumcise your son?

🞎 1 = Yes

🞎 0 = No

1. How much do you agree or disagree with the following statement, “Circumcision is viewed favorably by my friends and family” *[check one]*:

🞎 1 = Strongly agree

🞎 2 = Agree somewhat

🞎 3 = Disagree somewhat

🞎 4 = Strongly disagree

1. How much do you agree or disagree with the following statement, “I believe that among my friends and family men and boys are going for circumcision” *[check one]*:

🞎 1 = Strongly agree

🞎 2 = Agree somewhat

🞎 4 = Disagree somewhat

🞎 5 = Strongly disagree

1. In your understanding, which is true about male circumcision and the risk of HIV infection for men: *[Read responses 1-4 then choose one]*

🞎 1 = Completely protects a man from getting HIV from a woman

🞎 2 = Partially protects a man from getting HIV from a woman

🞎 3 = Does not affect a man’s chances of getting HIV from a woman

🞎 4 = Increases a man’s chances of getting HIV from a woman

🞎 5 = Unsure

***OPV-2 Group > 60 days of Life***

1. **Before** this interview right now, has anyone told you personally that your son [name] could be circumcised before 60 days of age? *[check one]*

🞎 1 = Yes

🞎 0 = No

🞎 2 = Not Sure

1. **Before** this interview right now, has anyone offered circumcision for your son [name] before 60 days of age? *[check one]*

🞎 1 = Yes *(go to a)*

🞎 0 = No *(skip to 57)*

🞎 2 = Not Sure *(skip to 57)*

1. If **YES**, where? *[check all that apply]*

🞎 1 = At my home

🞎 2 = At a hospital/clinic: (specify which):_________________________________

🞎 3 = Religious (specify): _____________________________________________

🞎 4 = Other (specify):_________________________________________________

🞎 5 = Not sure

1. If **YES**, who offered circumcision for your baby? *[check all that apply]*

🞎 1 = Domiciliary Midwife

🞎 2 = Community Health Worker

🞎 3 = Nurse, clinical officer or doctor at Ahero sub-District Hospital

🞎 4 = Nurse, clinical officer or doctor at Jaramogi Oginga Odinga Hospital

🞎 5 = Nurse or doctor at other health facility (*specify*):________________________

🞎 6 = Religious practitioner

🞎 7 = Other (*specify*): _________________________________________________

1. If **YES**, when? *[check all that apply]*

🞎 1 = Before this pregnancy

🞎 2 = During this pregnancy but before delivery

🞎 3 = At delivery / later the day of delivery or day after delivery

Between delivery and now

🞎 4 = At the child welfare clinic a week or less after birth

🞎 5 = At a vaccination visit

🞎 6 = At a visit because baby was sick

🞎 7 = Other visit (specify):_______________________________________

🞎 8 = Today at this health facility, before hearing about the “AccuCirc Study”

🞎 9 = Only now from staff from the “AccuCirc Study”

🞎 10 = Other (specify): _______________________________________________

🞎 11 = Not sure

1. **If YES,** were you personally for or against circumcising this baby?

🞎 1 = In favor

🞎 0 = Against

🞎 2 = Not sure

1. **If YES,** who was the primary person who made the decision not to circumcise your son? *[****check only one****]*

🞎 1 = Mother of infant (respondent)

🞎 2 = Father of infant

🞎 3 = Both of the infant’s parents equally

🞎 4 = Other *(specify relationship to respondent)*:

1. **If YES,** according to the views of the primary decision maker, what were all the reasons for choosing NOT to circumcise?

*[****A:*** *DO NOT read list of answers –****check all that apply****. Probe 🡺 Any other reason?]*

*[****B:*** *Read list of answers –****check all that apply****]*

**A B**

🞎 🞎 1 = Pain

🞎 🞎 2 = Bleeding

🞎 🞎 3 = Infection

🞎 🞎 4 = Injury to the penis

🞎 🞎 5 = Death from circumcision

🞎 🞎 6 = Going against cultural tradition

🞎 🞎 7 = It is better to wait until the boy is older

🞎 🞎 8 = The father was against it

🞎 🞎 9 = The baby was unwell

🞎 🞎 10 = The mother was unwell / tired after birth

🞎 🞎 11 = No particular reason

🞎 🞎 12 = Not sure

🞎 🞎 13 = Other (specify): ________________________________________

1. **If YES,** according to the primary decision maker, what was the single most important reason for choosing NOT to circumcise the baby? *[Read respondent’s answer[s] from previous question–****check only one****]*

🞎 1 = Pain

🞎 2 = Bleeding

🞎 3 = Infection

🞎 4 = Injury to the penis

🞎 5 = Death from circumcision

🞎 6 = Going against cultural tradition

🞎 7 = It is better to wait until the boy is older

🞎 8 = The father was against it

🞎 9 = The baby was unwell

🞎 10 = The mother was unwell / tired after birth

🞎 11 = No particular reason

🞎 12 = Not sure

🞎 13 = Other (specify): ________________________________________

1. Circumcision is only available to babies before two months of age. If we had offered circumcision for your baby before he turned two months, how likely is it you would have taken it up? *[check one]*

🞎 1 = Unlikely

🞎 2 = Neither likely nor unlikely

🞎 3 = Likely

🞎 4 = Not sure

1. How likely or unlikely are you to take your son for circumcision in the future? *[check one]*

🞎 1 = Unlikely

🞎 2 = Neither likely nor unlikely

🞎 3 = Likely

🞎 4 = Not sure

1. If you had another baby boy, would you want him to be circumcised? *[check one]*

🞎 1 = Yes

🞎 0 = No, I would want him to remain uncircumcised *(skip to 60)*

🞎 2 = Not sure *(skip to 60)*

1. If **YES**, at what age? *[check one]*

🞎 1 = Birth to eight weeks

🞎 2 = 9 weeks to <1 year old

🞎 3 = 1 to 9 years old

🞎 4 = 10 to 17 years old

🞎 5 = 18 years or older

🞎 6 = Any age

🞎 7 = Not sure

***Circumcision Group ≤ 60 Days of Life***

1. Where were you offered circumcision for your son *[name]*? *[check all that apply]*

🞎 1 = At home

🞎 2 = At a hospital/clinic: (specify which):_________________________________

🞎 3 = Religious (specify): _____________________________________________

🞎 4 = Other (specify):_________________________________________________

🞎 5 = Not sure

1. Who offered circumcision for your baby? *[check all that apply]*

🞎 1 = Domiciliary Midwife

🞎 2 = Community Health Worker

🞎 3 = Nurse, clinical officer or doctor at Ahero sub-District Hospital

🞎 4 = Nurse, clinical officer or doctor at Jaramogi Oginga Odinga Hospital

🞎 5 = Nurse or doctor at other health facility (*specify*):________________________

🞎 6 = Religious practitioner

🞎 7 = Other (*specify*): _________________________________________________

1. When was circumcision offered? *[check all that apply]*

🞎 1 = Before this pregnancy

🞎 2 = During this pregnancy but before delivery

🞎 3 = At delivery / later the day of delivery or day after delivery

Between delivery and now

🞎 4 = At the child welfare clinic a week or less after birth

🞎 5 = At a vaccination visit

🞎 6 = At a visit because baby was sick

🞎 7 = Other visit (specify):_______________________________________

🞎 8 = Today at this health facility, before hearing about the “AccuCirc Study”

🞎 9 = Only now from staff from the “AccuCirc Study”

🞎 10 = Other (specify): _______________________________________________

🞎 11 = Not sure

1. Are you personally for or against circumcising this baby?

🞎 1 = In favor

🞎 0 = Against

🞎 2 = Not sure

1. Who was the primary person who made the decision to circumcise your son? *[****check only one****]*

🞎 1 = Mother of infant (respondent)

🞎 2 = Father of infant

🞎 3 = Both of the infant’s parents equally

🞎 4 = Other *(specify relationship to respondent)*:__________________________________

1. A according to the views of the primary decision maker, what were all the reasons for choosing circumcision for your baby?

*[****A:*** *DO NOT read list of answers –****check all that apply****. Probe 🡺 Any other reason?]*

*[****B:*** *Read list of answers –****check all that apply****]*

**A B**

🞎 🞎 1 = Protection against HIV/STI

🞎 🞎 2 = Protection against Urinary Tract Infection (UTI)

🞎 🞎 3 = Penile hygiene / cleanliness

🞎 🞎 4 = Improved cosmetic appearance of the penis

🞎 🞎 5 = Less pain than when done later

🞎 🞎 6 = It is safer than when done later

🞎 🞎 7 = Religious reason

🞎 🞎 8 = Cultural reasons

🞎 🞎 9 = No particular reason

🞎 🞎 10 = Not sure

🞎 🞎 11 = Other (specify): ________________________________________

1. According to the primary decision maker, what was the single most important reason for choosing circumcision for your baby? *[Read respondent’s answer[s] from previous question–****check only one****]*

🞎 1 = Protection against HIV/STI

🞎 2 = Protection against Urinary Tract Infection (UTI)

🞎 3 = Penile hygiene / cleanliness

🞎 4 = Improved cosmetic appearance of the penis

🞎 5 = Less pain than when done later

🞎 6 = It is safer than when done later

🞎 7 = Religious reason

🞎 8 = Cultural reasons

🞎 9 = No particular reason

🞎 10 = Not sure

🞎 11 = Other (specify): ________________________________________

1. Please provide a contact number or contact information, in case we need to ask you any follow-up questions
2. Phone number: ______________________________________
3. Phone owner (name): ______________________________________
4. Other contact information: ______________________________________
5. **If you provided consent for us to contact the father of the baby**, please give a contact number or contact information for the father, so we can reach him for an interview

*[Check the consent form to confirm whether the participant gave consent to contact the father of the infant.]*

1. Phone number: ______________________________________
2. Phone owner (name): ______________________________________
3. Other contact information: ______________________________________

*[Read:* Thank you for your time. Those are all the questions I have for you. Do you have any questions for me right now?]

*[To be answered by the interviewer:]*

1. *Primary language of interview*

*🞎 1 = English*

*🞎 2 = DhoLuo*

*🞎 3 = Kiswahili*

1. *End Time*

| **:** |
| --- |

1. *Interviewer code*

|  |  |
| --- | --- |

1. *Notes: _________________________________________________________________________________________________________________________________________________________________________________________________________________________________________________________________________________________________________________________________________________________________________________________________________________________________________________________________*_
